# The inflammatory architecture reflects the effects of pharmacological and genetic interventions on resolution of TLR2-mediated inflammation

**DOI:** 10.3389/fimmu.2025.1633348

**Published:** 2025-10-17

**Authors:** Clara Kramer, Sandra Pierre, Nicole Zander, Anja Kolbinger, Blerina Aliraj, Andreas Weigert, Klaus Scholich

**Affiliations:** ^1^ Institute of Clinical Pharmacology, Goethe University Frankfurt, Frankfurt, Germany; ^2^ Institute of Biochemistry I, Goethe University Frankfurt, Frankfurt, Germany; ^3^ Department for Immunity of Inflammation, Mannheim Institute for Innate Immunoscience (MI3), Medical Faculty Mannheim, Heidelberg University, Mannheim, Germany; ^4^ Fraunhofer Institute for Translational Medicine and Pharmacology (ITMP), Frankfurt, Germany; ^5^ Fraunhofer Cluster of Excellence for Immune-Mediated Diseases (CIMD), Frankfurt, Germany

**Keywords:** inflammatory architecture, macrophages, neutrophils, TLR2, high-content imaging and analysis

## Abstract

**Introduction:**

Immune cells form defined pro- and anti-inflammatory regions around a pathogen during an innate immune response. These include, in Toll-like receptor (TLR)-2-induced inflammation, a core region containing the pathogen, an adjacent pro-inflammatory (PI) region and a surrounding anti-inflammatory (AI) region. Interventions targeting specific immune cells or signaling pathways disrupt this architecture and affect the resolution of inflammation. Here, we investigated, which changes in the inflammatory architecture may favor an increased resolution of inflammation.

**Methods:**

Immune cell networks and defined inflammatory regions were detected by high content imaging in an inflammation model induced by the TLR2 agonist zymosan. Resolution of inflammation was determined using thermal hypersensitivity.

**Results:**

Elimination of neutrophil recruitment using antibody depletion or GPR40-deficient mice had little effect on formation of the inflammatory structure or resolution of inflammation, as determined by the duration and strength of thermal hypersensitivity. High content imaging and FACS analysis showed that other phagocyting immune cells compensated for the loss of neutrophils in pathogen phagocytosis. In contrast, G2A-deficient mice, which exhibit enhanced resolution of zymosan-induced hypersensitivity, have reduced macrophage recruitment and polarization as well as a shift in the inflammatory architecture towards anti-inflammation. Importantly, the reduction of M1-like macrophage polarization without reduction of macrophage numbers by the JAK1/2 inhibitor baricitinib was not sufficient to alter the inflammatory structure or resolution of inflammation.

**Discussion:**

Combined with previously published results in the same inflammation model, we find that a strong decrease or increase of the PI region negatively affects resolution of inflammation, whereas a moderate decrease of 30-50% is associated with in part strongly enhanced resolution of TLR2-mediated inflammation.

## 1 Introduction

During inflammation, immune cells form different microenvironments that represent the functions they perform in these areas. In the recent years, especially the tumor microenvironment has been the subject of extensive research, which has led to a good understanding of the cells involved, their interactions and the molecules involved in the formation and maintenance of this microenvironment (1, 2). This research has been able to take advantage of the fact that tumors are often relatively easily distinguished from surrounding healthy tissue. The formation and maintenance of microenvironments in other inflammatory responses is much less understood due to the high mobility of pathogens and their often diffuse and undetectable distribution. A potential solution to this problem is the use of immobilized pathogens that can used as reference point within the tissue to define their microenvironment. This approach allows immune cell networks to be delineated and mapped relative to the reference point, providing a comprehensive understanding of the immune response within the tissue (3). Zymosan is a commonly used inducer of Toll-like receptor 2 (TLR2)-mediated local inflammation, that is arrested at the site of injection due to its particulate structure. Using fluorescently labeled zymosan, its localization can be precisely determined and the position of immune cells relative to the zymosan can be defined (3–7). TLR2-mediated immune responses are relevant, since TLR2 is nearly ubiquitously expressed in immune and non-immune cells, initiating acute innate immune responses directed against a wide range of pathogens including gram-positive bacteria, protozoa and viruses and thereby allowing to induce an inflammation in a wide range of tissues (8, 9).

To determine the microenvironments in this inflammation model high-content immunohistochemistry is especially useful. It is capable of visualizing an unlimited number of antibodies on the same tissue sample and allows single-cell phenotyping and bioinformatics analysis of the number, phenotype and localization of the involved immune cells (3, 10, 11). This technology has been shown to help identifying the mechanism of action of drugs (4) as well as predicting patient prognosis (12, 13) and therapy resistance (14). Moreover, it allowed the identification of the inflammatory architecture of zymosan-induced inflammation, showing a core region containing the pathogen, neutrophils and M1-like macrophages. This core region is surrounded by a pro-inflammatory (PI) region dominated by M1-like macrophages, which in turn is surrounded by an anti-inflammatory (AI) region, which is characterized by the presence of M2-like macrophages (3–5). These three regions form within 24 hours after zymosan injection and persist until zymosan removal is complete (3).

Interventions targeting the immune response in this model showed distinct effects on the inflammatory architecture, whereby the likelihood of two cell types to be direct neighbors can be used to define a relative distance (3–5). For example, treatment with the non-steroidal-anti-inflammatory drug (NSAID) meloxicam reduced proinflammatory responses leading to shrinkage of the PI region, as determined by the relative distance between zymosan and M2-like macrophages (4). In contrast, genetic depletion of mast cells, which fulfill an anti-inflammatory role in the zymosan model, caused expansion of the PI region (5). Importantly, the pharmacological or genetic interventions tested so far for their influence on the inflammatory regions also impaired resolution of inflammation, regardless of whether they caused the PI region to shrink (meloxicam, eosinophil depletion) (3, 4) or to expand (mast cell depletion) (5).

Here, we aimed to test the hypothesis that the PI region is indicative of effects on the resolution of a TLR2-mediated inflammation and to investigate how a strong interference with the immune response, which does not alter the resolution of inflammation, affects the inflammatory regions. Therefore, we depleted neutrophils, which are the dominant immune cell type in the inflamed tissue during the onset of zymosan-induced inflammation (6, 15, 16), but do not affect the resolution of zymosan-induced inflammation when depleted (15). In addition, we used G2A knockout mice to test the effect of a resolution-promoting intervention on the inflammatory structure during the zymosan-induced inflammation. In these mice, macrophage migration to the core region is impaired, resulting in a selective decrease in the number of M1-like macrophages (7, 17).

## 2 Material and methods

### 2.1 Mice

Male C57BL/6 mice (6–8 weeks) were provided by Janvier (Le Genest, France). GPR40 knockout mice with C57BL/6N background (6–8 weeks) were previously described (7, 18). Frozen paw tissue from previously published experimentation with G2A knockout mice (The Jackson Laboratory, Bar Harbor, ME) were used for MELC analyses (7). Mice were treated according to the International Association for the Study of Pain guidelines. All the ethics guidelines for investigations in conscious animals were observed and the procedures were approved by the local ethics committee (Regierungspräsidium Darmstadt). All animals had free access to water and food. The room temperature (23 ± 0,5 °C) and light (7:00 am and 7:00 pm) were controlled. The animals were randomized for the experiments. Inflammation was induced by injection of 10 µl Zymosan A (3mg/ml in PBS, Merck, Darmstadt, Germany) subcutaneously into the plantar side of one hind paw. For neutrophil depletion, 500 µg Ly6G antibody clone 1A8 InVivoPlus™ from Bio X Cell (Dartmouth, NH) was injected intraperitoneal (i.p.) 24 hours before the zymosan injection. The same amount of 500 µg rat IgG2α isotype clone 2A3 (Bio X Cell, Dartmouth, NH) was used as control and was injected 24 hours before zymosan injection as well. Baricitinib was administered orally (10 mg/kg body weight in carboxymethyl cellulose (CMC).

### 2.2 Thermal hypersensitivity and edema formation

In all behavioral tests the experimenter was unware of the treatment of the animals. The edema measurements were done at the indicated times after zymosan injection in one hind paw. Edema volumes were measured with a 37140 plethysmometer from Ugo Basile (IITC Life Science, Woodland Hills, CA) by immersion of the mouse hind paw (19). The zymosan-induced thermal hypersensitivity was determined with the Hargreaves test using an IITC Plantar Analgesia Meter (Hargreaves test; IITC Life Science, Woodland Hills, CA, USA) and the maximal temperature was at 32°C with a cut off time at 20 seconds.

### 2.3 Resolution scores

To score the effect of interventions on the resolution of zymosan-induced hypersensitivity published data (3–7) were reanalyzed for the time points 24, 48 and 72 hour after zymosan injection. A first scoring (Score 1) was applied to significant (Two Way ANOVA, Bonferroni *post hoc* test) changes in the paw withdrawal latency (PWL) compared to the respective control mice. It was scored with 1 when the intervention increased PWL toward baseline, 0 when there was no change and -1 when the PWL decreased. A second score (Score 2) was calculated in case the PWL returned to baseline earlier in the intervention group than the control group an additional score of +2 was applied per day (One Way ANOVA, Bonferroni *post hoc* test). In case the PWL returned to baseline earlier in the control group score of -2 was applied per day. Both scores were added to generate the final resolution score.

### 2.4 Multi-epitope-ligand-carthography

Multi-epitope-ligand cartography (MELC) is an automated immunohistological imaging method that can be used to visualize high numbers of antibodies on the same sample (3, 10). Briefly, 10 µm tissue sections on silanized coverslips were fixed with 4% paraformaldehyde in PBS for 10 minutes, permeabilized with 0.1% Triton X-100 in PBS for 10 min, and blocked with 3% bovine serum albumin (all from Merck, Darmstadt, Germany) in PBS for 1 hour. Tissue samples were imaged using a DMi8 microscope (Leica Microsystems, Wetzlar, Germany) and a HC PL FLUOTAR L 20x/0,040 CORR PH1 objective with a cooled sCMOS camera (2048 × 2048 pixels). The sample was then incubated with up to 3 antibodies, each labeled with different bleachable fluorescence-tags, and washed with PBS. Phase-contrast and fluorescence images were collected, the fluorescence signals were bleached and post-bleaching images were recorded. The process was repeated until all antibodies were imaged. For data analysis, the post-bleaching images were subtracted from the following fluorescence image. The antibodies used on the MELC system are listed in the **Supporting Data S1**.

### 2.5 Image analysis

All grayscale antibody channel images were edited with ImageJ v1.53q (National Institutes of Health [NIH], Bethesda, MD, USA) to remove background fluorescence, noise and artifacts for the analyses. CellProfiler (v4.2.1) is an open-source software for analyzing cell images. It was used to create a cell mask and lightning corrections (20). CellProfiler is necessary for additional illumination correction and the generation of a cell mask for single-cell segmentation using the propidium iodide (cell nuclei) and CD45 (cluster of differentiation). The cell mask was imported in histoCAT (v1.7.3) (11) with the corresponding antibody channel images. All images, except images used for single-cell mask generation, were z-score normalized and used for Barnes-Hut t-SNE (BH t-SNE) (21) and PhenoGraph analysis (22) in histoCAT. PhenoGraph defines cell clusters based on single-cell mask and marker colocalization (k set between 15 and 30). BH t-SNE scatter plot was overlaid with a colored PhenoGraph cluster map and clusters were classified as cell types based on their marker expression. To calculate the relative number of cells per cell type, the number of objects per cluster was normalized to the total number of objects in the cell mask. The z-score normalized images were exported to FlowJo software v10.8.1. Pairwise interactions between cell phenotypes were calculated for each cell and their neighbors in a distance of four pixel and were imported into Cytoscape (v3.8.2) to produce dual-centered neighborhood networks showing relative distances to the defined centers (23).

### 2.6 FACS analysis

Cell isolation and preparation from blood and paws was done as described previously (1, 9, 19). Briefly, inflamed paws were cut into <1 mm^3^ pieces and incubated for 45 minutes at 37°C in 500 µl lysis buffer (3 mg/ml Collagenase (from Clostridium histolyticum Typ IA, Merck, Darmstadt, Germany) in RPMI 1640 medium). Lysis was stopped by addition of 5 ml 10% FBS in DMEM. The cells were passed through a cell strainer (70 µm) and incubated in erythrocyte lysis (ACK) buffer for 5 minutes at room temperature. The cells were centrifuged, fixed and then permeabilized at 4°C for 10 minutes using the BD Cytofix/Cytoperm Fixation/Permeabilization Kit (BD Biosciences, Heidelberg, Germany). Unspecific binding was blocked by incubation with 60 µl of 2% Fc-blocking reagent Mouse BD Fc Bloc (BD Pharmingen, NJ, USA) in PBS for 10 minutes at 4°C. Antibodies (**Supplementary Table S1**) were incubated for 20 minutes at 4°C. Samples were acquired with a flow cytometry system (FACSymphony A5 Cell Analyzer; BD Biosciences, Heidelberg, Germany) and analyzed by using FlowJo software v10 (BD Biosciences, Heidelberg, Germany). Unstained controls and fluorescence minus one (FMO) controls were used to establish the gating strategy. Cell fragments and cell clusterings were excluded based with FCS/SSC gating based on the particle sizes. Additional Live dead staining was not included. According to this gating strategy the signals considered as live, single cells comprised around 60% of all signals after isolation of cells from paws. To detect phagocyting cells pH-sensitive pHrodoTM Red Zymosan A BioParticles conjugate (Invitrogen, Eugene, Oregon, USA) was used for injection and cells isolated from an inflamed paw injected with unlabeled zymosan were used as FMO control.

### 2.7 RNA sequencing

Bone marrow cells were isolated from femur and tibia from the hind legs of wild type and GPR40-knockout mice. The cells were passed through a cell strainer (70 µm), incubated in erythrocyte lysis (ACK) buffer for 5 minutes at room temperature, centrifuged, washed and resuspended in PBS-0,5% BSA. Unspecific binding was blocked by incubation with 60 µl of 2% Fc-blocking reagent Mouse BD Fc Bloc on ice. FACS sorting was performed using a FACS Diva (BD Biosciences) based on granularity and Ly6G^high^ expression (anti-Ly6G APC-cy7 (1A8)). RNA isolation, sequencing, quantification of mapped reads and differential transcript expression analysis was done by GenXPro GmbH (Frankfurt, Germany). Pathway analysis of the results was done using EnrichR (24, 25) and String (26) mRNA Sequencing data are available in the GUDe database (https://gude.uni-frankfurt.de/handle/gude/591).

### 2.8 Statistical analysis

Statistically significance was calculated using one-way or two-way analysis of variance (ANOVA) by GraphPad Prism v9.0.1. For *post hoc* analysis Bonferroni correction for multiple comparisons was used. Comparisons between two groups were performed by unpaired two-tailed Student’s t-test with Welsch´s correction.

## 3 Results

### 3.1 Neutrophil depletion does not affect the PI region or resolution of TLR2-mediated inflammation

**Figure 1:**
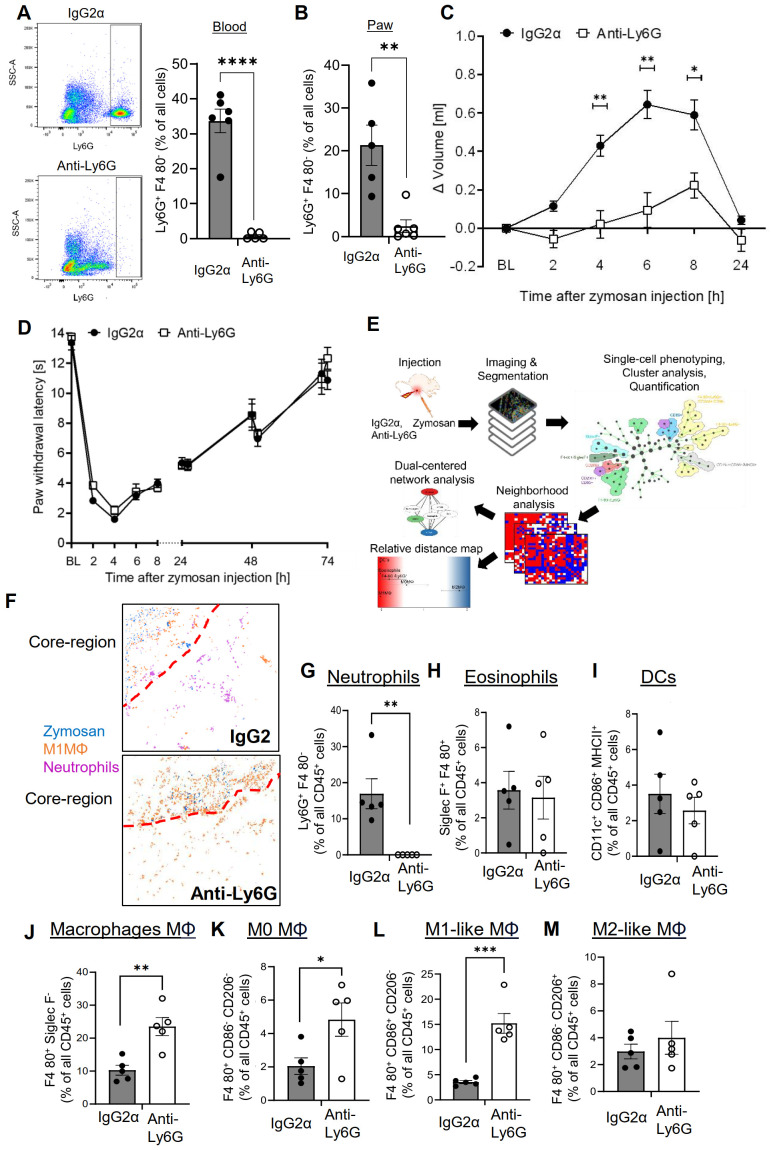
Neutrophil depletion decreases edema formation but not thermal hypersensitivity. **(A, B)** FACS-analysis of neutrophils in blood (panel A) and paws (panel B) 24 hours after zymosan injection in mice having received IgG2α (500 µg, i.p.) or anti-Ly6G (500 µg, i.p.) antibodies. Data are shown as mean ± SEM (n=5-6); multiple unpaired t-test ** P<0.01, **** P<0.0001. **(C, D)** Edema formation (Panel C) and thermal hypersensitivity (panel D) of mice injected with IgG2α or anti-Ly6G antibodies. Data are shown as mean ± SEM (n=6); Two-way ANOVA, Bonferroni * P<0.05, ** P<0.01. **(E)** Workflow for the bioinformatics analysis of the MELC images. **(F)** Representative images of cell clusters for neutrophils and M1-like macrophages in regard to zymosan in paws 24 hours after zymosan injection in mice treated with IgG2α- or anti-Ly6G-antibodies. Red dotted lines depict the border of the core region. **(G-M)** Quantification of MELC data 24 hours after zymosan injection for the number of neutrophils **(G)**, eosinophils **(H)**, DCs **(I)**, all macrophages **(J)**, M0-like macrophages **(K)**, M1-like macrophages **(L)** and M2-like macrophages **(M)** in mice treated with IgG2α- or anti-Ly6G-antibodies. Data are shown as mean with SEM (n=5); unpaired t-test * P<0.05, ** P<0.01, *** P<0.001.

Previously it was shown that neutrophil depletion using the anti-Ly6G/Ly6C antibody clone GR1 strongly decreased zymosan-induced edema formation without affecting extent or resolution of hypersensitivity (15). Here we used the more specific anti-Ly6G antibody clone 1A8, which reduced the number of neutrophils by >90% in blood and inflamed paws 24 hours after zymosan injection (**Figures 1A, B** and **Supplementary Figure S2A, B**). Notably, we used the anti-Ly6G/Ly6C antibody GR1 for FACS and immunohistochemical detection of neutrophils. This antibody recognizes neutrophils without interference from the 1A8 antibody, which was used for neutrophil depletion (**Supplementary Figure S3A**). In agreement with previous results (15), the zymosan-induced edema formation was strongly decreased in the depleted mice (**Figure 1C**) whereas onset and resolution of zymosan-induced thermal hypersensitivity were not affected by neutrophil depletion (**Figure 1D**). To determine the effect of neutrophil depletion on the inflammatory architecture, the high-content imaging technology MELC was used with an established panel of 29 antibodies recognizing immune cells and non-immune cells (**Supplementary Table S1**) (3–5, 27). For single cell phenotyping, cluster analysis, quantification and neighborhood analysis the previously established workflow was used (**Figure 1E**) (3–5). Visual fields for MELC imaging were selected to cover the zymosan-containing region (approximately 30% of the total image) and the neighboring areas. MELC analysis of paws 24 hours after zymosan injection confirmed a reduction of the neutrophil number in the tissue by >90% (**Figures 1F, G** and **Supplementary Figure S3B**). Neutrophil depletion did not change the number of cells in clusters representing eosinophils and dendritic cells (DCs) in the observed areas of the inflamed paws (**Figures 1H, I**) whereas the total number of macrophages significantly increased (**Figure 1J**). Further bioinformatics analysis of the MELC data showed that cell clusters representing M1-like macrophages (Siglec F^–^/F4-80^+^/CD86^+^/CD206^–^) and M0 macrophages (Siglec F^–^/F4-80^+^/CD86^+^/CD206^+^) were responsible for this increase, while the number of M2-like macrophages (Siglec F^–^/F4-80^+^/CD86^-^/CD206^+^) did not change (**Figures 1K–M**). As reported previously, no innate lymphoid cells, B cells, T cells and NK cells were detected in this area of the tissue during this phase of the zymosan-induced inflammation (3, 4).

**Figure 2:**
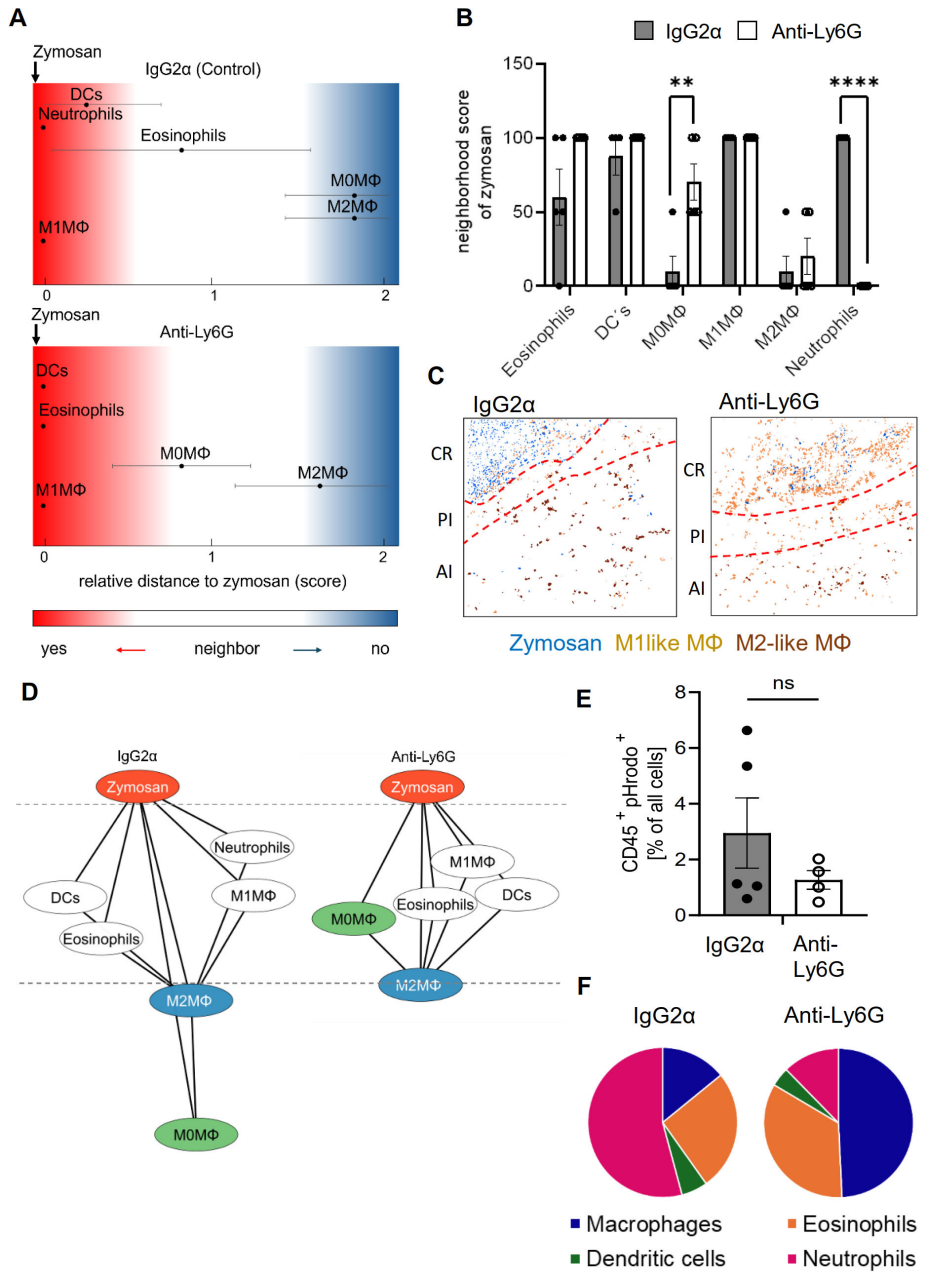
Neutrophil-depletion does not alter the relative size of the PI region. **(A, B)** Zymosan centered relative distance mapping (panel A) and histogram (panel B) for various immune cell types in mice treated with IgG2α or anti-Ly6G antibodies 24 hours after zymosan injection. Data shown as mean (n=5) ± SEM; multiple unpaired t-test; ** P<0.01, *** P<0.001. **(C)** Representative images of clusters for zymosan, M1-like and M2-like macrophages in paws 24 hours after zymosan injection in mice treated with IgG2α- or anti-Ly6G-antibodies. Red dotted lines depict the border of the core region **(D)** Dual-centered network visualization of the combined cellular neighborhoods of zymosan and M2-like macrophages in paws from mice treated with IgG2α or anti-Ly6G antibodies 24 hours after zymosan injection. **(E, F)** FACS analysis of all pHrodo^+^ CD45^+^ cells (panel E) and percentage of pHrodo^+^ cell types (panel F) in IgG2α and Anti-Ly6G pretreated mice 4 hours after zymosan injection. Data are shown as mean of n = 4-5; unpaired T-test, ns, not significant.

To determine the effect of neutrophil depletion on the inflammatory structure, the likelihood of cells neighboring each other was compared to randomized cell distributions (15). For visualization of the neighborhood of zymosan, linear distance maps of the relative distance between zymosan and the identified cell clusters were generated based on scores ranging from 0 (direct neighbors) to 2 (no neighbors). Based on these scores, the relative distance between zymosan and M2-like macrophages can be used to define the size of the PI region (3–5). Importantly, neutrophil depletion did not change the size of the PI region (**Figures 2A–C**). Neutrophil depletion also led to surprisingly small effects on the relative distance to zymosan for most of the other immune cell types (**Figures 2A, B**). Only CD86/CD206 double positive M0 macrophages showed a significant decrease in their relative distance to zymosan (**Figures 2A, B**), suggesting a reduced pressure of the microenvironment in the PI region on macrophages to polarize toward M1-like macrophages. Also the dual-centered network visualization, which combines the cellular neighborhoods of zymosan and M2-like macrophages, and allows a clearer presentation of the distribution of the immune cells in the tissue, showed, with the exception of the M0 macrophages, no relevant changes in the network localization of the different cell types (**Figure 2D**). One of the major functions of neutrophils in inflammation is the removal of pathogens by phagocytosis. Therefore, we determined which cell types are involved in zymosan phagocytosis in our model using pHrodo-zymosan, whose pH-sensitive fluorescent label is activated upon internalization into lysosomes. FACS analysis showed that the number of pHrodo-zymosan^+^ immune cells did not change after neutrophil depletion (**Figure 2E**), with an increased proportion of pHrodo-zymosan^+^ macrophages (**Figure 2F**; **Supplementary Figures S4A, B**). This increase in phagocytic macrophages was not detected in MELC analysis (**Supplementary Figures S5A–D**), which might be due to the incomplete coverage of the core region in the field of visions. Since the timely pathogen clearance was unaffected in neutrophil-depleted mice as reflected by the normal resolution of zymosan-induced thermal hypersensitivity, it can be speculated that the phagocytic capacity of macrophages and other phagocytic immune cells, which is otherwise used to clear apoptotic neutrophils, can be redirected toward phagocytosis of zymosan.

### 3.2 GPR40 deficiency does not affect the size of the PI region or thermal hyperalgesia

**Figure 3:**
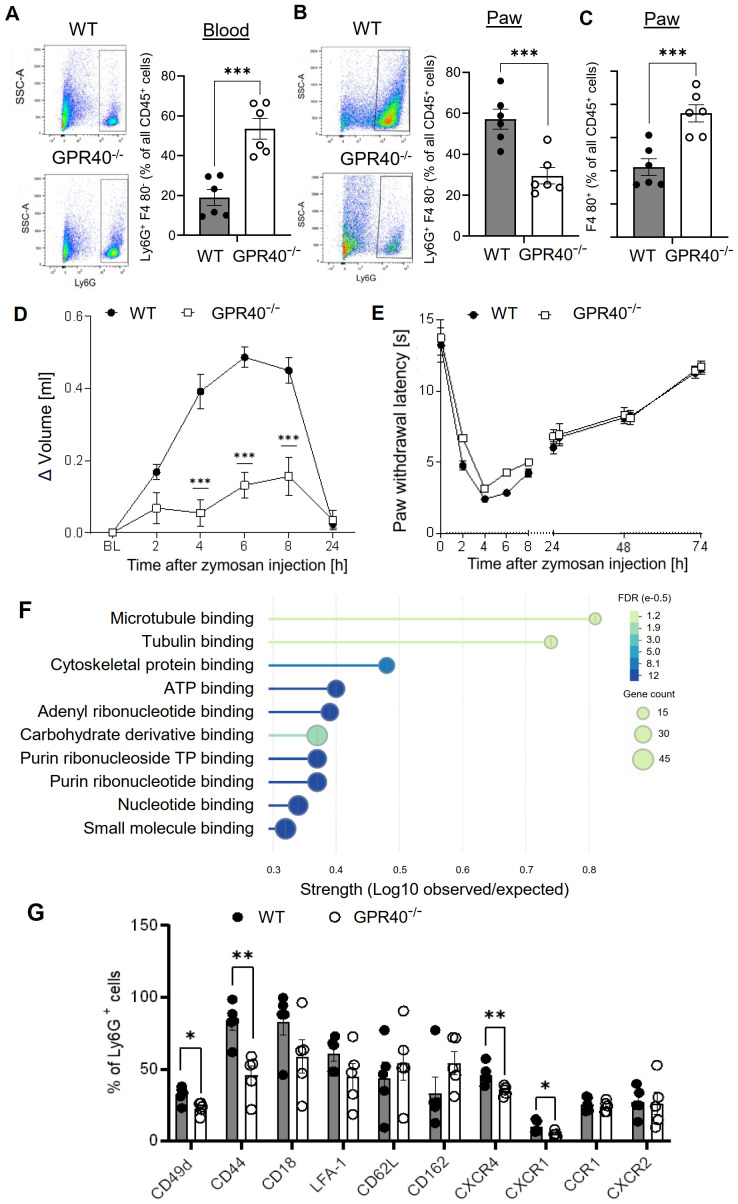
Neutrophil recruitment is abolished in GPR40-knockout mice. **(A-C)** FACS-analysis of neutrophils (Ly6G^+^ F4-80 ^-^) (panel A, B) and macrophages (F4-80^+^) (panel C) in blood (panel A) and paws (panel B, C) of wild type and GPR40-knockout mice 24 hours after zymosan injection. Data are shown as mean ± SEM (n=5-6). Student´s t-test, ** P< 0.01, *** P< 0.001. **(D, E)** Zymosan-induced edema formation (panel D) and thermal hypersensitivity (panel E) in wild type (WT) and GPR40-knockout mice. Data are shown as mean ± (n=6). Two-way ANOVA/Bonferroni, *** P<0.001. **(F)** String analysis for molecular function of mRNA expression levels in bone marrow neutrophils from naïve wild type and GPR40-knockout mice. Strength is the ratio between annotated number of proteins and the expected annotated number in a random network of the same size. False discovery rate (FDR) is color coded and gene count is depicted as circle size. **(G)** FACS analysis of integrins and chemokines expression on neutrophils in the blood of untreated wild type and GPR40-knockout mice. Data shown as mean ± (n=5). Two tailed Students T-test * P <0.05; ** P <0.01.

Notably, neutrophil depletion results in their phagocytosis by macrophages, which might alter the course of inflammation. Therefore, GPR40 (FFAR1) knockout mice were tested as a second model to interfere with neutrophil functions. The G protein-coupled receptor (GPCR) GPR40 is highly expressed in neutrophils and positively regulates neutrophil functionality (28–30). FACS analysis showed that although neutrophil numbers were upregulated in the blood of GPR40 knockout mice (**Figure 3A**; **Supplementary Figure S6A**), neutrophil recruitment to zymosan-injected paws was reduced in the GPR40 knockout mice (**Figure 3B**). Analogous to the findings in the antibody-mediated neutrophil depletion model, FACS analysis also showed increased numbers of macrophages in the inflamed paw (**Figure 3C**), suggesting a similar compensatory mechanism. As seen in the antibody-mediated neutrophil depletion model, the edema formation was strongly reduced in GPR40 knockout mice (**Figure 3D**) while the resolution of the zymosan-induced thermal hypersensitivity was not altered (**Figure 3E**). To investigate the mechanisms underlying the decreased neutrophil recruitment in GPR40 knockout mice, we determined the mRNA expression levels by RNAseq of neutrophils isolated from the bone marrow of wild type and GPR40 knockout mice. Pathway analyses using the EnrichR and String databases showed a prominent decrease in the expression of genes associated with the tubulin cytoskeleton, including the downregulation of tubulin-α-1c and the kinesins Kif4, Kif11, Kif15 and Kif22 (**Figure 3F**; **Supplementary Figure S6B** and **S9**). Since the tubulin cytoskeleton is required for endosomal transport involved in receptor transport to the plasmamembrane (31), we performed FACS analysis of neutrophils isolated from the blood to study the expression of 10 chemokine and integrin receptors, that mediate neutrophil recruitment to inflamed tissue. We found a significant downregulation of CD44, CD49d, CXCR1 and CXCR4 (**Figure 3G**; **Supplementary Figure S4A**), which have been shown to mediate bone marrow egress of neutrophils, neutrophil adhesion to endothelial cells, neutrophil tissue recruitment and endothelial transmigration of neutrophils (32–36). Thus, the data show that loss of GRP40 abolishes neutrophil recruitment during zymosan-induced inflammation, which appears to be based on a multifactorial perturbation of cytoskeletal functions and receptor surface expression.

**Figure 4:**
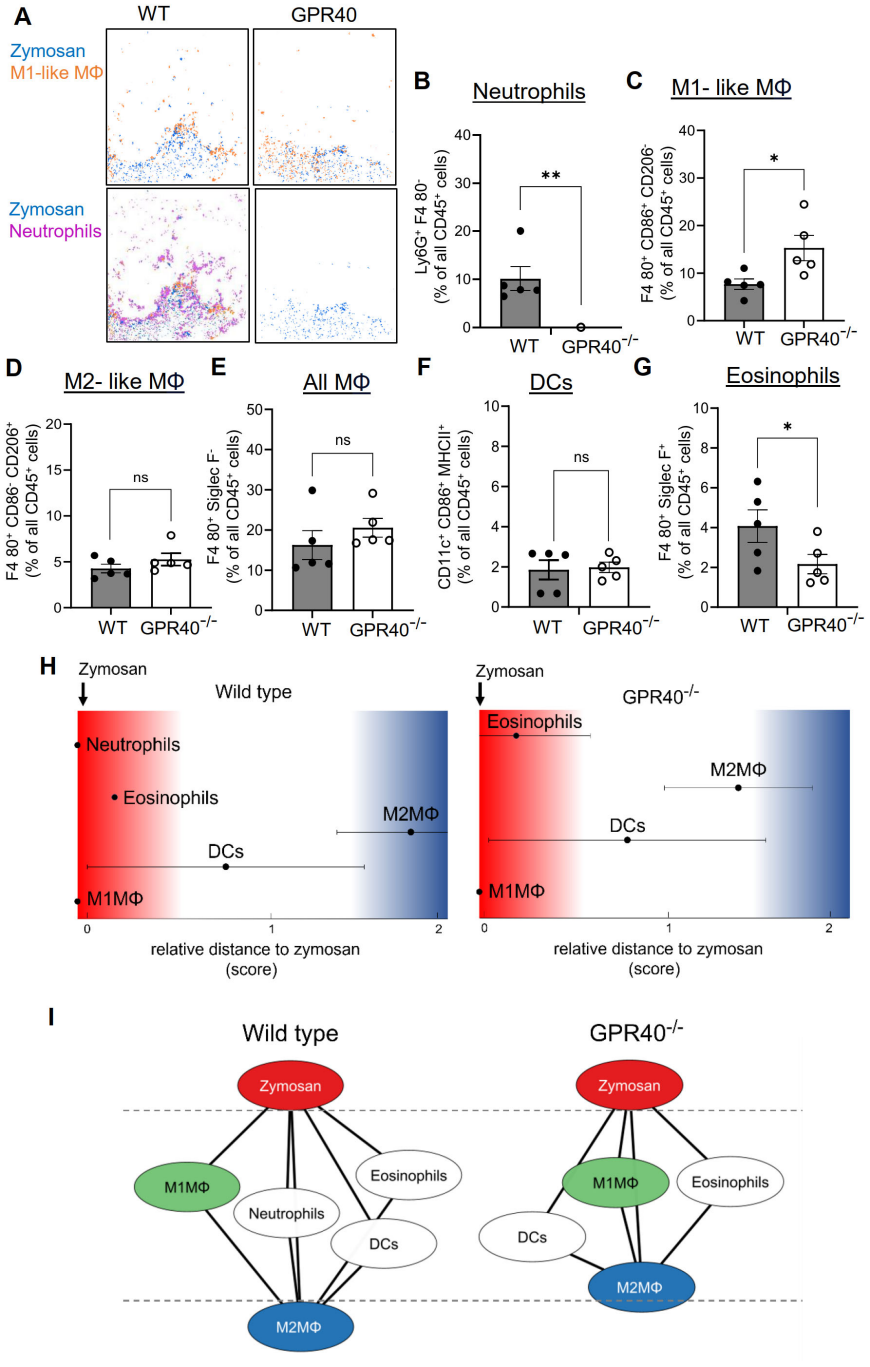
The relative size of the PI region does not change in GPR40-knockout mice. **(A)** Representative images of cell clusters for neutrophils and M1-like macrophages in regard to zymosan in paws 24 hours after zymosan injection in wild type and GPR40-knockout mice. **(B-G)** MELC-analysis of the number of neutrophils **(B)**, M1-like macrophages **(C)**, M2-like macrophages **(D)**, all macrophages **(E)**, DCs **(F)** and eosinophils **(G)** in paws from wild type and GPR40-knockout mice 24 hours after zymosan injection. Data are shown as the mean ± SEM (n=5); unpaired t-test * P<0.05, ** P<0.01. **(H)** Zymosan-centered relative distance analysis of paws 24 hours after zymosan injection in wild type (control) and GPR40-knockout mice. Data shown as mean (n=5 mice). ± SEM. **(I)** Dual-centered network visualization of the cellular neighborhoods of zymosan and M2-like macrophages 24 hours after zymosan injection in wild type (control) and GPR40-knockout mice.

MELC analysis of the inflamed paws confirmed the nearly complete absence of neutrophils 24 hours after zymosan injection (**Figures 4A, B**). Notably, the difference in the neutrophil numbers between the MELC and the FACS analyses shown in **Figure 3B** can be attributed to the presence of contaminating blood in the tissue preparations used for the FACS analysis. Fittingly, this was not an issue after antibody-mediated neutrophil depletion, since the blood was also depleted of neutrophils. As seen for antibody-mediated neutrophil depletion an increase of cells in clusters representing M1-like macrophages was observed (**Figure 4C**), suggesting a compensatory upregulation similar to the results for antibody-mediated neutrophil depletion. The number of M2-like macrophages, all macrophages and DCs did not change (**Figures 4D–F**), while the number of eosinophils decreased by approximately 50% (**Figure 4G**), suggesting a previously unknown role for GPR40 in their recruitment to the inflamed tissue. Most importantly, the neighborhood analysis showed only a minor shift of M2-like macrophages toward the zymosan, which did not reach significance (**Figures 4H, I**). Taken together, the data show that neutrophil depletion, either mediated by antibodies or due to GPR40-deficiency, does not significantly affect the size of the PI region as defined by the relative distance between zymosan and M2-like macrophages. Accordingly, also the resolution of the zymosan-induced thermal hypersensitivity was not affected.

### 3.3 G2A-deficiency decreases the PI region and enhances resolution of hyperalgesia

**Figure 5:**
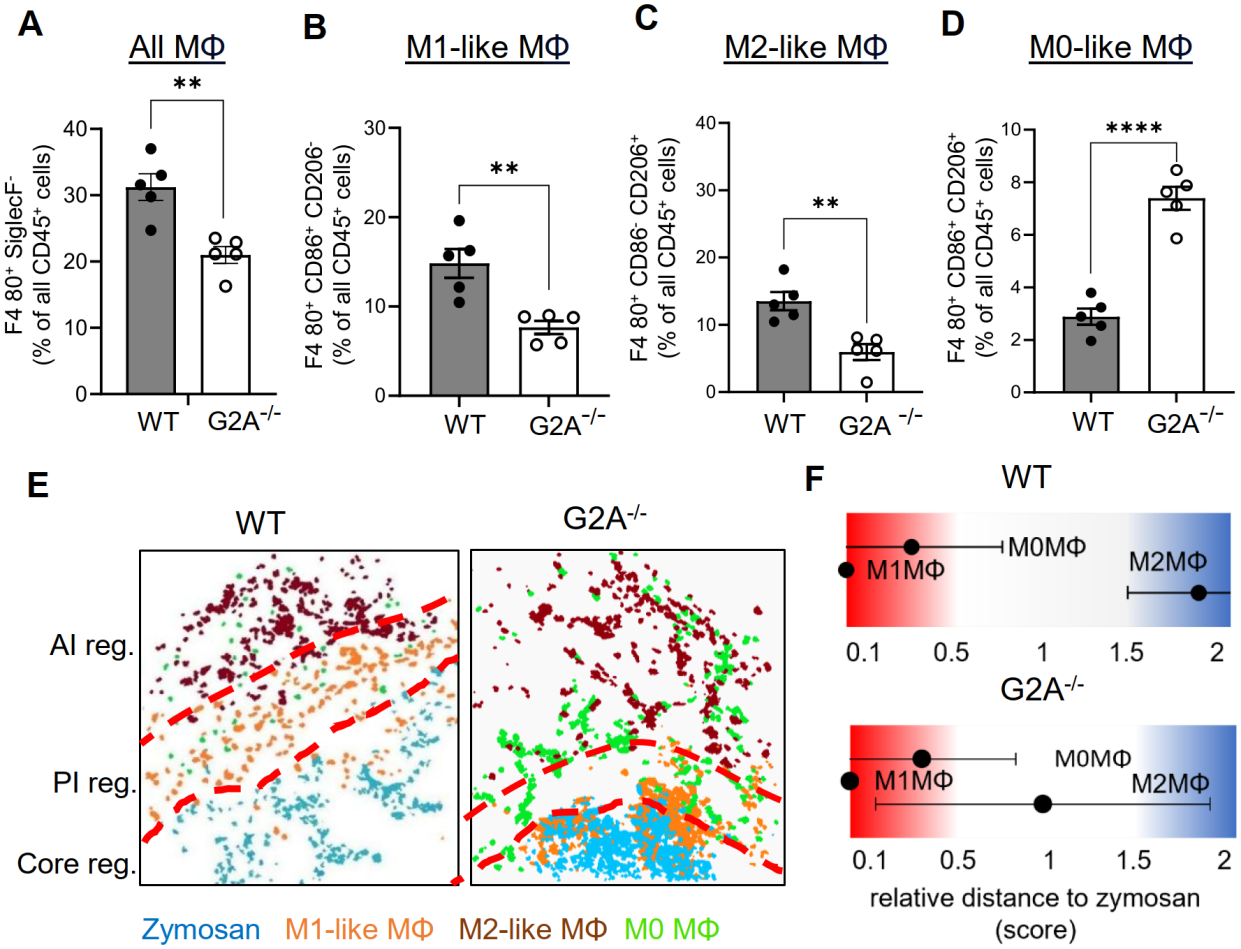
The number of M1-like macrophages and the relative size of the PI region decreases in G2A knockout mice. **(A-D)** MELC-analysis of the numbers of all macrophages **(A)** M1-like macrophages **(B)**, M2-like macrophages **(C)** and M0-like macrophages **(D)** in paws from wild type and G2A-knockout mice 24 hours after zymosan-injection. Data shown as mean (n=5 mice) ± SEM; unpaired T-test ** P<0.01, **** P<0.0001. **(E)** Representative images of cell clusters for M1-like, M2-like and M0 macrophages in regard to zymosan in paws 24 hours after zymosan injection in wild type and G2A-knockout mice. Red dotted lines depict the borders between the inflammatory regions. **(F)** Zymosan-centered relative distance analysis of paws in wild type and G2A-knockout mice 24 hours after zymosan injection. Data shown as mean (n=5 mice) ± SEM.

The GPCR G2A (GPR132) is expressed on macrophages and mediates efferocytosis of apoptotic neutrophils by increasing chemotaxis toward apoptotic “find-me” signals (37–40). Accordingly, G2A deficiency causes reduced macrophage migration to the zymosan-containing core region, thereby reducing the M1-like macrophage numbers in this area, which results in an enhanced resolution of inflammation (7). To determine the effect of the G2A-deficiency on the PI region, we performed MELC analyses using inflamed paws from wild type and G2A-deficient mice 24 hours after zymosan injection. Consistent with previous publications (7, 38, 39), MELC analysis showed in G2A knockout mice a significant decrease of the number of macrophages in the observed area (**Figure 5A**). Also, the number of M1-like (**Figure 5B**) and M2-like macrophages decreased (**Figure 5C**). Interestingly, the number of CD206/CD86 double positive M0 macrophages increased, suggesting an overall decreased polarization pressure on macrophages by their microenvironment (**Figure 5D**). To investigate the effect of the locally altered recruitment and polarization of macrophages on the regional structure of the inflammation, we performed a neighborhood analysis. We found an increased likelihood of M2-like macrophages to neighbor zymosan whereby the relative distance between zymosan and M2-like macrophages decreased by 50% (**Figures 5E, F**). Thus, in G2A deficient mice, the strongly enhanced resolution of inflammation (7) is accompanied by decreased numbers and polarization of macrophages as well as shrinkage of the PI region by 50%.

### 3.4 Baricitinib shifts macrophage polarization without altering the PI region or resolution of zymosan-induced hyperalgesia

**Figure 6:**
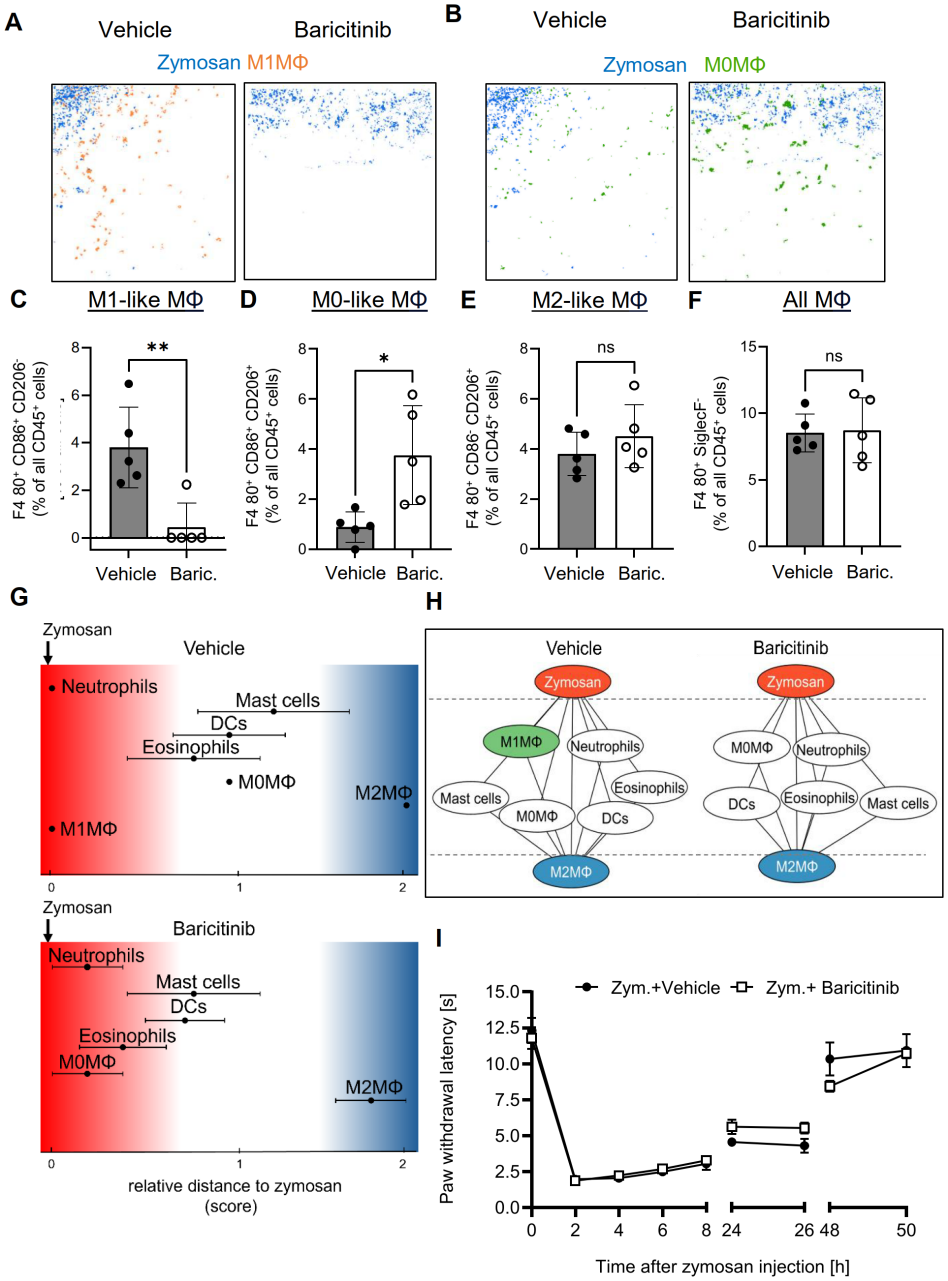
Baricitinib abolished polarization toward M1-like macrophages, but does not affect the PI region or resolution of inflammation. **(A, B)** Representative images of cell clusters for M1-like **(A)** and M0 macrophages **(B)** in regard to zymosan in paws 24 hours after zymosan injection in mice treated with vehicle or baricitinib. **(C-F)** MELC-analysis of the numbers of M1-like **(B)**, M0-like **(C)** and M2-like macrophages **(D)**, and all macrophages **(E)** of paws from mice treated with vehicle or Baricitinib (10 mg/kg) 24 hours after zymosan-injection. Data are shown as mean ± SEM (n=6); unpaired t-test * P<0.05; ** P<0.01. **(G)** Zymosan-centered relative distance analysis of immune cell in mice 24 hours after zymosan injection treated with vehicle or baricitinib (10 mg/kg). Data are shown as mean (n=5 mice) ± SEM. **(H)** Dual-centered network visualization of the cellular neighborhoods of zymosan and M2-like macrophages in vehicle and baricitinib-treated mice 24 hours after zymosan injection. **(I)** Zymosan-induced thermal hypersensitivity of vehicle and baricitinib-treated animals. Data are shown as mean ± SEM (n=6); Two-way ANOVA/Bonferroni.

Since G2A-deficiency increased the resolution of inflammation and was accompanied by decreased M1-like macrophage numbers, we investigated whether inhibition of M1-like macrophage polarization is sufficient to decrease the PI region and to promote the resolution of inflammation. Therefore, we used the immunosuppressive Janus kinase (JAK) 1/2 inhibitor baricitinib, which blocks among others the M1-like promoting cytokines IL-6 and INFγ (41). MELC analysis showed that baricitinib treatment abolished M1-like macrophage polarization and caused an increase of the number of the CD86/CD206 double-positive M0 macrophages (**Figures 6A–D**). Notably, neither the number of M2-like macrophages nor the total number of macrophages was altered (**Figures 6E, F**). Similarly, there was no significant effect on the number of DCs, eosinophils, neutrophils or mast cells seen in the observed inflammatory area (**Supplementary Figures S7A–D**). Neighborhood and network analyses showed a shift of M0 macrophages toward the zymosan, which is likely due to the reduced polarization of M0 macrophages toward M1-like macrophages, which are thereby reduced in their numbers (**Figures 6G, H**). Most importantly, the neighborhood analysis showed no change in the likelihood of zymosan and M2-like macrophages being neighbors, demonstrating that the treatment does not alter the size of the PI region (**Figures 6G, H** and **Supplementary Figure S7E**). Finally, baricitinib treatment had no effect on the resolution of thermal hypersensitivity (**Figure 6I**), demonstrating that preventing the polarization of M1-like macrophages alone is not sufficient to promote the resolution of inflammation.

### 3.5 Localization of M2-like macrophages mirror changes in the resolution of zymosan-induced hyperalgesia

Next, we calculated the effect of the 4 different interventions presented in this study and reevaluated the data from previously published data for eosinophil depletion (3), meloxicam treatment (4), mast cell deficiency (5) and TP knockout mice (6) for the relative size of the PI region as determined by the likelihood of zymosan and M2-like macrophages. These values were plotted against a score describing the resolution of inflammation, which was calculated by scoring the change in time required for the zymosan-induced hypersensitivity to return to baseline as well as the change in paw withdrawal latency as compared to the respective control treatments.

**Figure 7:**
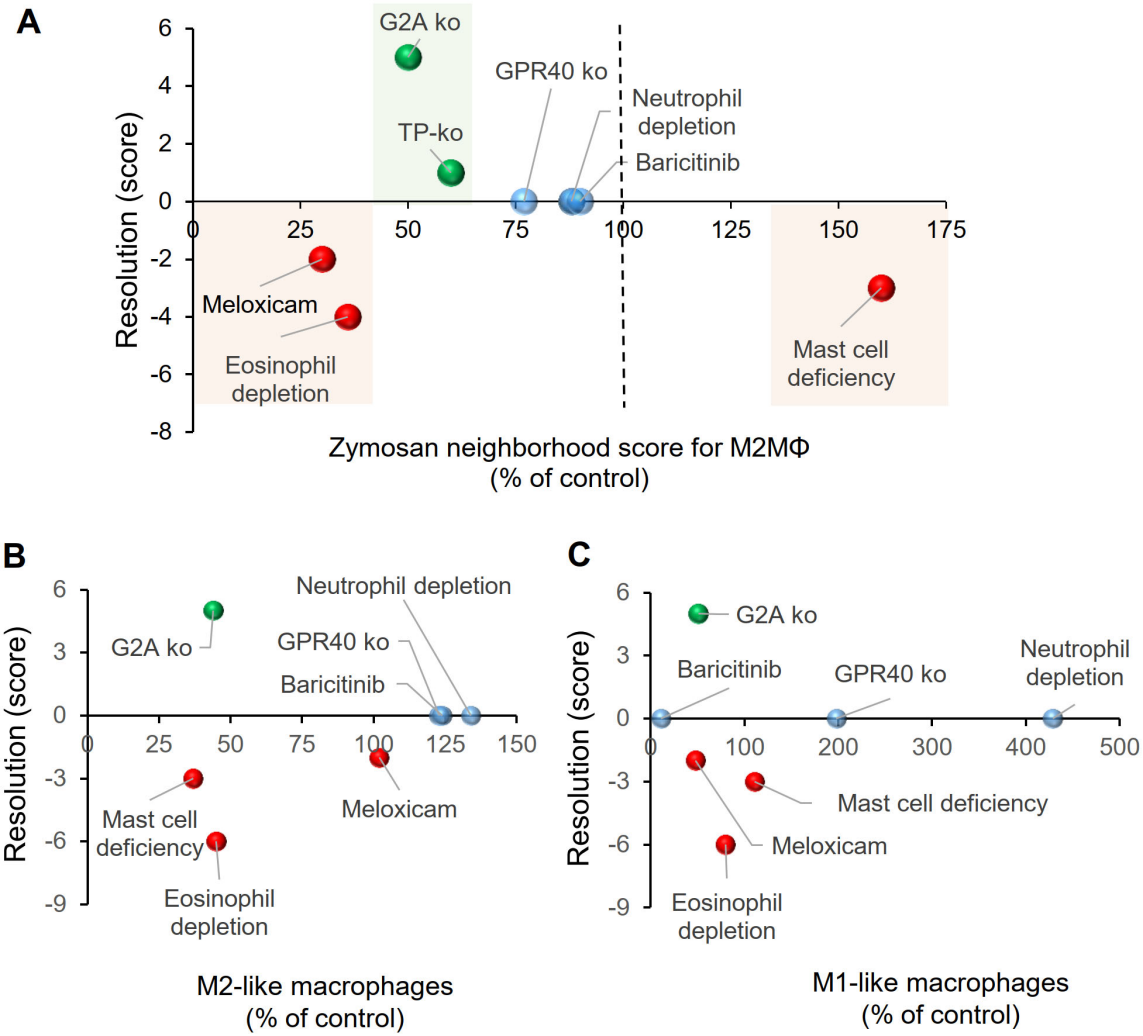
Relationship between the relative size of the PI region and the resolution score for zymosan-induced hypersensitivity. **(A)** Effect of pharmacological or genetic interventions on resolution zymosan-induced hypersensitivity and the size of the PI region 24 hours after zymosan injection. Resolution scores were calculated as described in the Methods section. The sizes of the PI regions were determined by the relative distance between zymosan and M2-like macrophages and are shown as percent of the respective control treatments. **(B, C)** Effect of pharmacological or genetic interventions on resolution of zymosan-induced hypersensitivity and the number of M2-like (panel B) or M1-like (panel C) macrophages determined in MELC analyses.

We found that a small reduction of the size of the PI region of less than 30%, as seen in GPR40 knockout mice, after neutrophil depletion and after baricitinib treatment, correlates with a normal resolution of inflammation (**Figure 7A**; **Supplementary Table S8**). In contrast, strong changes of the size of the PI region, as observed for eosinophil depletion and meloxicam treatment (>65% decrease of the PI region) (3, 4) or in mast cell-deficient mice (50% increase of the PI region) (5), were associated with a delayed resolution of inflammation (**Figure 7A**). The only interventions leading to improved resolution of inflammation, were the G2A- and TP-knockout, which showed a decrease of 50-60% for the PI region (**Figure 7A**). Notably, no dependence of the resolution score on the number of M1-like or M2-like macrophages in the observed area was seen (**Figures 7B, C**). In summary, the data show that the size of the PI region mirrors pro- and anti-inflammatory effects of interventions, whereby small or no effects on the size of the PI region do not affect resolution of a TLR2-mediated inflammation. A moderate decrease of the size of the PI region is associated with promotion of resolution while stronger changes are associated with a reduced resolution. These effects mirrored the net effect on the immune response and was not clearly associated with changes in macrophage subtypes.

## 4 Discussion

During TLR2-mediated zymosan-induced innate inflammation three major inflammatory regions can be distinguished. These include the pathogen-containing core region, which is encircled by a PI region dominated by M1-like macrophages and an outer AI region containing M2-like macrophages (3–5). The boundaries of the central PI are defined on one side by the presence the pathogen and on the other side by the presence of M2-like macrophages (3–5). Because of its location between the core and AI regions, its size is influenced by the balance between the pro- and anti-inflammatory responses from the other two regions. The size of the PI region therefore reflects the net effect of an intervention targeting one or more components of the immune response. However, it is important to note that, unlike an epidermis, for example, the PI region is not a permanent structure with specific resident cells and stable borders. Rather, PI regions constantly migrate within tissue as they follow the shrinking core region. Similarly, repositioning the PI region shifts the AI region into areas previously occupied by the PI region. Therefore, it is expected that proinflammatory and anti-inflammatory mediators released in either region will directly or indirectly influence the shape and functionality of the other interconnected regions. consequently, the PI region, in particular, cannot be studied as an individual entity but rather must be interpreted in the context of the entire inflammatory architecture.

Importantly, although the size PI region is defined by the relative distance between zymosan and M2-like macrophages, the M1- or M2-like macrophages numbers did not correlate with the size of the PI region or the resolution of inflammation. For example, while eosinophil depletion delayed and G2A-deficiency promoted resolution, both interventions caused a similar decrease in the number of M1- and M2-like macrophage phenotypes. Also, abolishing M1-like macrophage polarization by baricitinib treatment did not affect resolution of inflammation, since M0 macrophages appear to be able to compensate for the loss of M1-like macrophages. Compensatory mechanisms also mitigate the impact of neutrophil depletion or GPR40 deficiency, underscoring the adaptable nature of the involved immune cell networks. This capacity enables effective responses to evolving conditions and external stimuli. However, one limitation of this study is that it is not possible to achieve a comprehensive description of gene expression patterns in various macrophage subpopulations using antibody-based high-content imaging technology. Therefore, the macrophages were separated into only three basic subpopulations (M0, M1-like, and M2-like macrophages), which does not fully represent the biological complexity of macrophages.

The dependence of the PI region on the pro- and anti-inflammatory responses of the neighboring core and AI regions, allows it to reflect the net effect of the interventions on the immune response. In this regard, major disturbances in the immune response lead to strong reduction or increase of the size of the PI region, which as consequence negatively impacts resolution of inflammation. This was observed during meloxicam treatment, which reduced strongly the PI region by inhibiting prostanoid synthesis, and also in eosinophil depletion and mast cell deficiency (3–5). The general suppression of the pro-inflammatory response in pathogen-induced inflammation is unfavorable for resolution of the inflammation, since resolution is directly dependent on the removal of pathogens and cell debris from apoptotic and necrotic cells (42, 43). Indeed, meloxicam treatment (4) and eosinophil depletion (3) reduced zymosan phagocytosis, causing a delayed resolution of inflammation. Conversely, mast cell deficiency increased the size of the PI region, suggesting a reduced anti-inflammatory response. However, this also led to decreased efferocytosis (27) and consequently negatively impacting resolution of inflammation (5). Therefore, it seems logical that a limited reduction in the pro-inflammatory response that does not interfere with pathogen clearance may promote resolution of inflammation by accelerating tissue regeneration processes. In line with this hypothesis, G2A-deficiency, which has been shown to promote resolution of inflammation, exhibited a 50% reduction in the PI region. However, further research with other pro-resolution modulators, such as specialized pro-resolving mediators (e.g. lipoxins, resolvins) or EP4 agonists (44) is needed to determine whether such moderate reduction in the PI region is indicative of the effects of resolution-promoting modulators.

The identification of the underlying mechanisms that mediate the effect of an intervention on the size of the PI region is complicated by the dependence on the balance and context-specific relevance of all pro- and anti-inflammatory mediators. In this regard, some pharmaceutical agents target specific inflammatory mediators that possess both multifaceted and occasionally contradictory functions within the inflammatory system. Meloxicam, for instance, inhibits the generation of several prostanoids, which can exhibit both pro- and anti-inflammatory functions (45). Accordingly, prostaglandin (PG) E_2_ has been found to inhibit eosinophil migration, while PGD_2_ promotes it (46, 47). Also, PGE_2_ is known to polarize macrophages toward M2-like phenotypes, while the prostanoids thromboxane (TX) A2 and PGD_2_ promote M1-like polarization (6, 48). Since PGE_2_, PGD_2_ and TXB2 are all synthesized during zymosan-induced inflammation, the functional relevance of these prostanoids depends on the tissue location of their synthesis as well the balance between each other and also with other inflammatory mediators, such as cytokines and chemokines. Similarly, the immunosuppressant JAK1/2 inhibitor baricitinib blocks signaling pathways connected to a diverse array of receptors and mediators. These include proinflammatory mediators such as IL-6 and IFNγ, as well as anti-inflammatory mediators, such as IL-4 and IL-10, which play a role in the zymosan model (3, 5, 27, 41). Therefore, the overall effect of baricitinib is observed, as some effects may be obscured by compensatory or redundant mechanisms.

In addition, most drug targets are expressed in multiple cell types affecting them in various ways. In this regard, GPR40 is expressed in neutrophils, endothelial cells and macrophages, evoking cell type-specific responses, such as enhanced neutrophil function, improved endothelial barrier function and M2-like macrophage polarization (28, 30, 49–51). The most dramatic effect observed in GPR40 knockout mice is a complete lack of neutrophil recruitment to the inflamed paw. Additionally, an increased polarization of macrophages toward M1-like phenotypes was seen. However, it is difficult to determine whether the increase in M1-like macrophages is due to an increased number of macrophages in the proinflammatory microenvironment of the core region or an GPR40-mediated intracellular effect on macrophage polarization (50, 51). Thus, the *in vivo* relevance of the various cellular functions in the different cell types, which are regulated by GPR40 in this specific inflammation model is still unclear.

In regard to the effect of neutrophil depletion on eosinophil functionality we showed previously that eosinophils play an important role in structuring the inflammatory answer to zymosan. Antibody-mediated eosinophil depletion strongly altered the inflammatory architecture so that a clear distinction between the PI and AI regions was no longer detectable. This led to decreased phagocytosis by macrophages, decreased polarization toward M2-like macrophage phenotypes and increased neutrophil recruitment (3). In the light of the small alterations of the inflammatory architecture in both models for neutrophil depletion, there is no strong indication for a significant effect on eosinophil functionality. Antibody-mediated neutrophil depletion did not significantly alter the eosinophil numbers, their localization or the percentage of eosinophil phagocytizing pHrodo-zymosan. In GPR40 deficient mice the number of eosinophils even decreased in the inflamed paw in GPR40-deficient mice whereby their general localization did not change. Also, in both models there were only minor effects on the inflammatory structure after neutrophil depletion.

Taken together, the characterization of the immune cell networks underlying the inflammatory architecture allows the identification of the impact of cells on the cellular network beyond their immediate cellular neighborhood, allowing a deeper understanding of the underlying pathomechanisms and mode of action of drugs. Conveniently, the same imaging datasets used to determine the inflammatory architecture can be used to gain mechanistic insights into the quantitative and qualitative effects on other cells, as well as to unravel compensatory mechanisms or effects on phagocytosis and efferocytosis, allowing new insights into the cellular networks that form the different regions and the control of the balance and interaction of these regions.

## Data Availability

The datasets presented in this study can be found in online repositories. The names of the repository/repositories and accession number(s) can be found in the article/**Supplementary Material**.
