# Supplementary material for: The inflammatory architecture reflects the effects of pharmacological and genetic interventions on resolution of TLR2-mediated inflammation
Source: Front Immunol. 2025 Oct 17;16:1633348. doi: 10.3389/fimmu.2025.1633348 (PMC12575189; doi:10.3389/fimmu.2025.1633348)
Supplement: Supplementary file 1 [file DataSheet1.pdf]

Table S1: Antibodies used for MELC and FACS analysis.

Target	Fluoro-chrome	Dilution	Company	Clone number	Order number
CCR10	PE	1:50	R+D Systems		FAB2815P
CD3	APC	1:100	Miltenyi Biotec	REAG1	130-109-838
CD4	AF488	1:100	Southern Biotech	L3T4	1540-02
CD8a	FITC	1:100	BD Pharmingen	53-6.7	553030
CD11b	FITC	1:400	BioRad	M1/70.15	MCA74F
CD11c	PE	1:100	Miltenyi Biotec	N418	130-122-952
CD19	FITC	1:100	BDPharmingen	1D3	557398
CD22	APC	1:100	Miltenyi Biotec	Cy34.1	130-102-576
CD27	PE	1:100	Miltenyi Biotec	REA499	130-114-166
CD31	PE	1:400	BD Biosciences	MEC13.3	553373
CD45	FITC	1:200	Miltenyi Biotec	30F11.1	130-116-535
CD41	FITC	1:50	AbDSerotec	MWReg30	MCA2245F
CD80	FITC	1:100	Biolegend	16-10A1	104706
CD86	PE	1:100	Biolegend	GL-1	105008
CD117	APC	1:50	Bioss		bs-10005R-Cy5
CD206	APC	1:100	Biolegend	C068C2	141708
CD183 (CXCR3)	PE	1:100	Miltenyi Biotec	REA724	130-111-087
CD194 (CCR4)	PE	1:100	Biolegend	2G12	131203
CD196 (CCR6)	PE	1:100	Biolegend	29-2L17	129803
Cytokeratin	AF488	1:800	eBiosciences	AE1/AE3	53-9003-82
CX3CR1	FITC	1:200	Biolegend	SA011F11	149020
CXCR7	APC	1:50	Biolegend	8F11-M16	331113
F4-80	PE	1:400	Biolegend	BM8	123110
Ly6C	APC	1:100	eBioscience	HK 1.4	17-5932-82
Ly6G-Gr1	PE	1:400	eBioscience	RB6-8C5	12-5931-83
Ly6G	APC	1:100	Biolegend	1A8	127614
MHC II	PE	1:100	Miltenyi Biotec	REA813	130-102-186
Propidium-Iodide		1:800	Sigma		P4170
Siglec F	PE	1:200	BD Bioscience	E50-2440	552126
**Antibodies used only for FACS Analysis**					
CD11c	BV711	1:50	Miltenyi		130-102-797
CD11b	BV605	1:50	Biolegend	M1/70	101237
CD45	AF700	1:50	Biolegend	30-F11	103128
CD86	PE	1:50	Biolegend	GL-1	105032
F4-80	FITC	1:50	Miltenyi	REA126	130-117-509
Ly6G	APC-Cy7	1:50	Biolegend	1A8	127624
Siglec F	PE-Vio770	1:50	Miltenyi	ES22-10D8	130-102-167

Figure S2

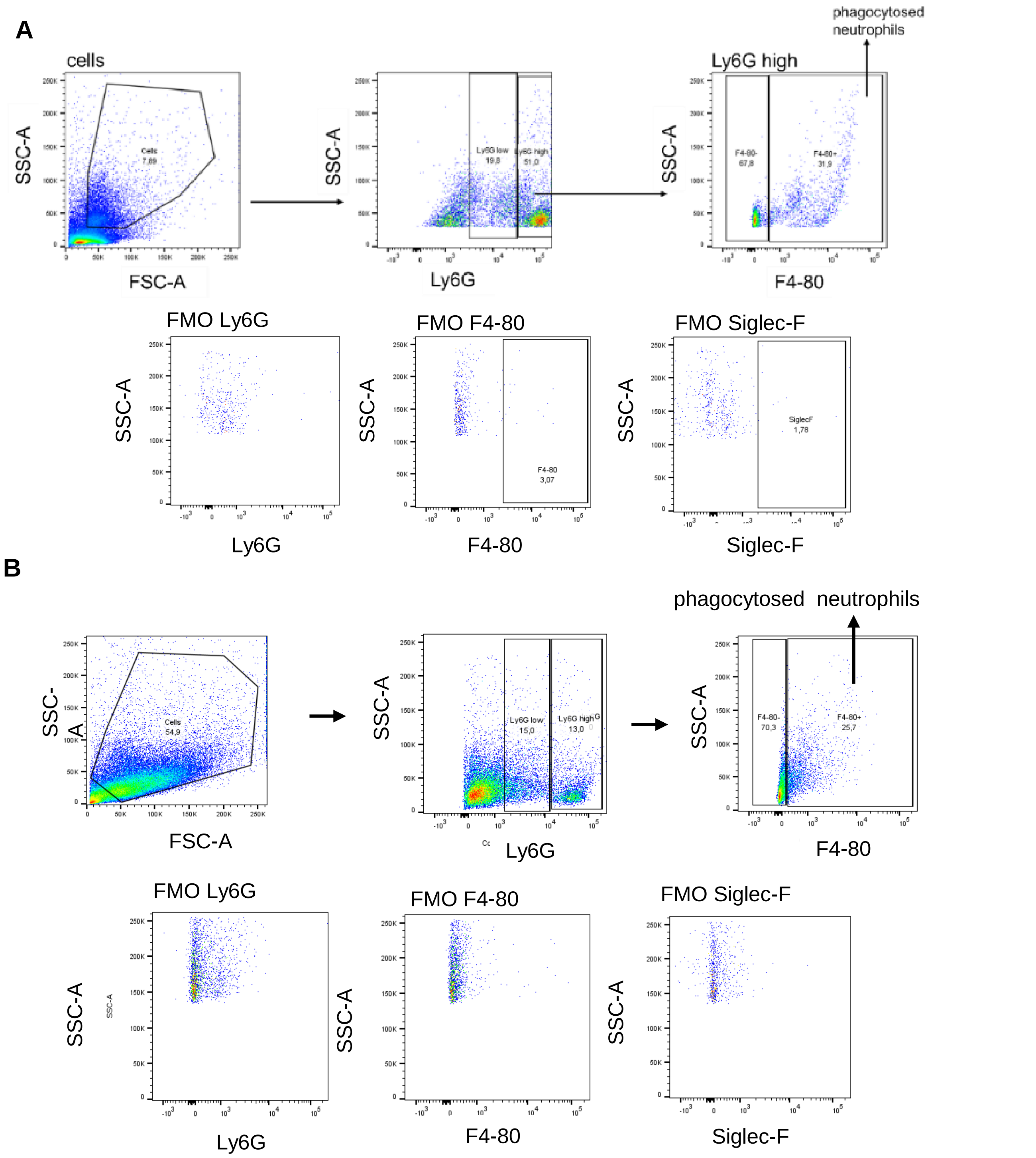

Figure S2: Binding of the anti-Ly6G antibody 1A8 does not interfere with GR-1 antibody binding. FACS gating strategy for neutrophils and macrophages in blood (A) and paws (B) 24 hours after zymosan-injection (10 µl, 3 mg/ml) in mice receiving the control antibody. Cells were first gated based on their side scatter area (SSC-A) and forward scatter area (FSC-A). Ly6G^{+}/F4-80^{-} cells were identified as neutrophils, F4-80^{+} were identified as macrophages. F4-80^{+}/Ly6G^{+} cells were defined as macrophages phagocyting apoptotic neutrophils. The lower panels show the FMO controls.

Figure S3

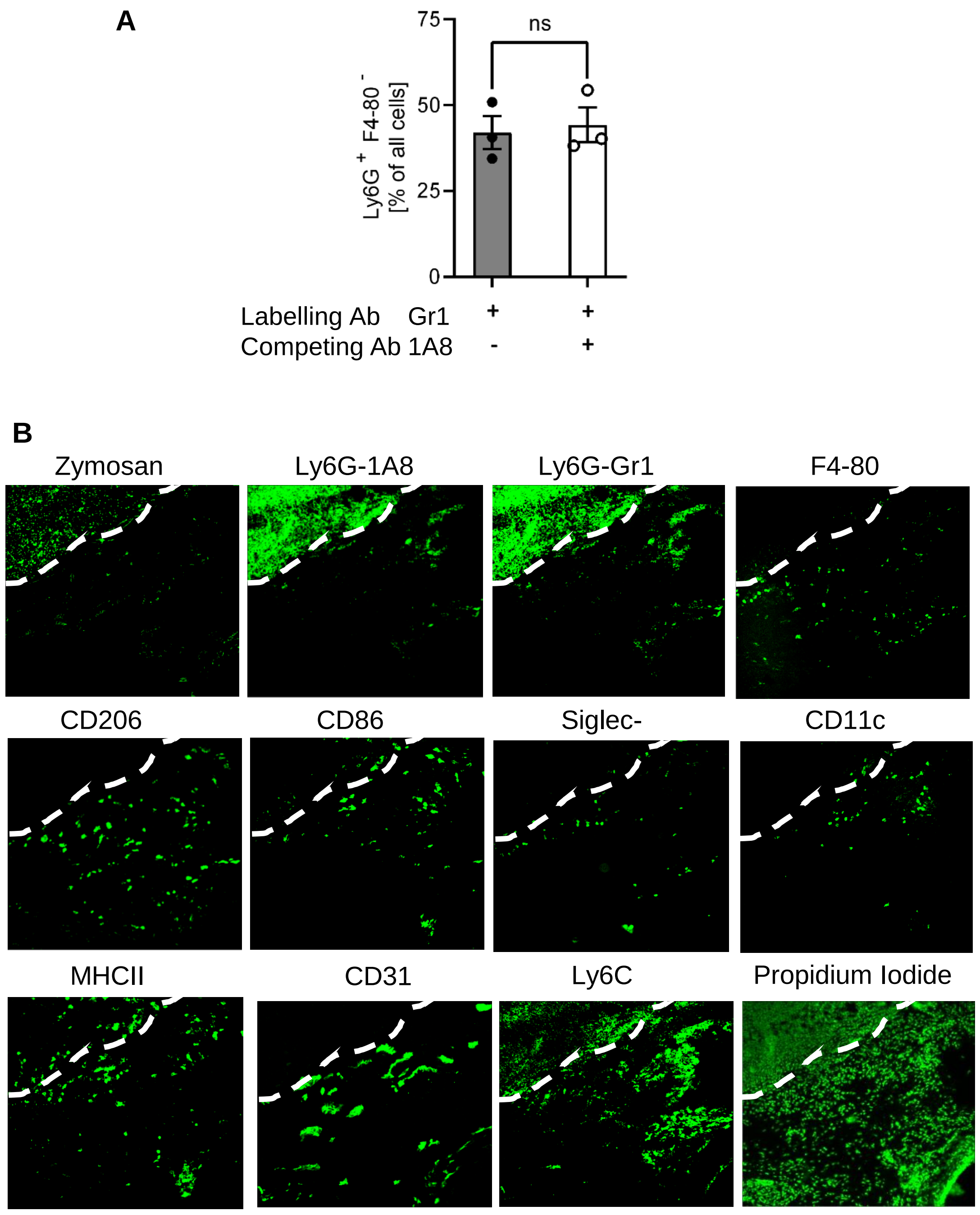

Figure S3: Binding of the anti-Ly6G antibody 1A8 does not interfere with GR-1 antibody binding. **(A)** FACS analysis of white blood cells from untreated control mice, which were stained with the Ly6G-antibody GR1 in absence or presence of the unlabelled Ly6G-antibody 1A8 in the staining mix. Data are shown as mean ± SEM (n=3); Two tailed t-test, ns = not significant. **(B)** Representative MELC images of inflamed paws 24 hours after zymosan injection from mice receiving control IgG2 antibody. The dotted lines depict the outline of the zymosan-covered area.

Figure S4

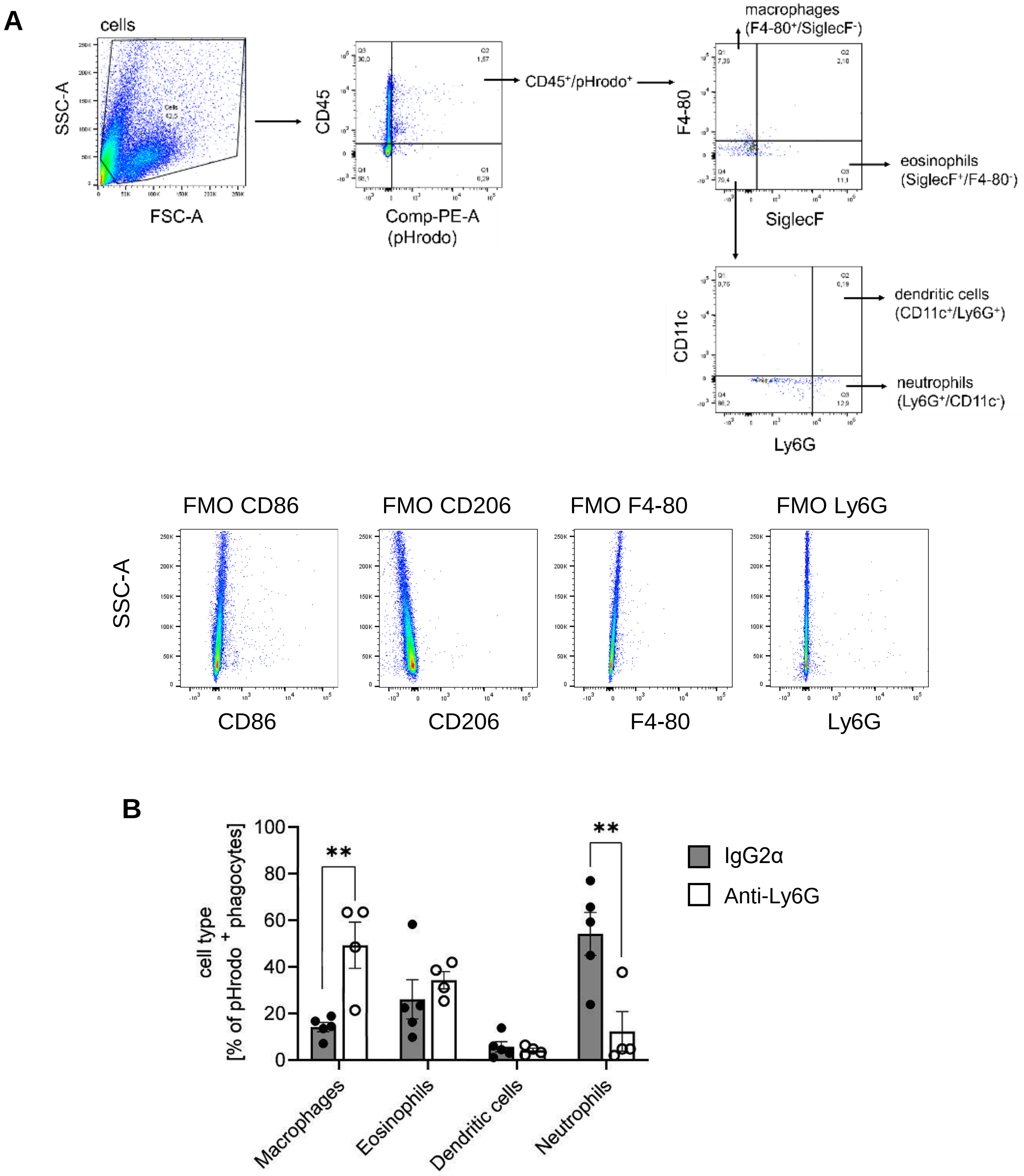

Figure S4: In vivo assay for phagocytosis of pHrodo zymosan.
(A) FACS gating für pHrodo+ immune cells applied in panel B.
(B) FACS-analysis of cell types of pHrodo^{+} immune cells in paws 4 hours after zymosan-pHrodo injection. Data are shown as the mean ± SEM (n= 4-5); multiple unpaired t-test ** P<0.01

Figure S5

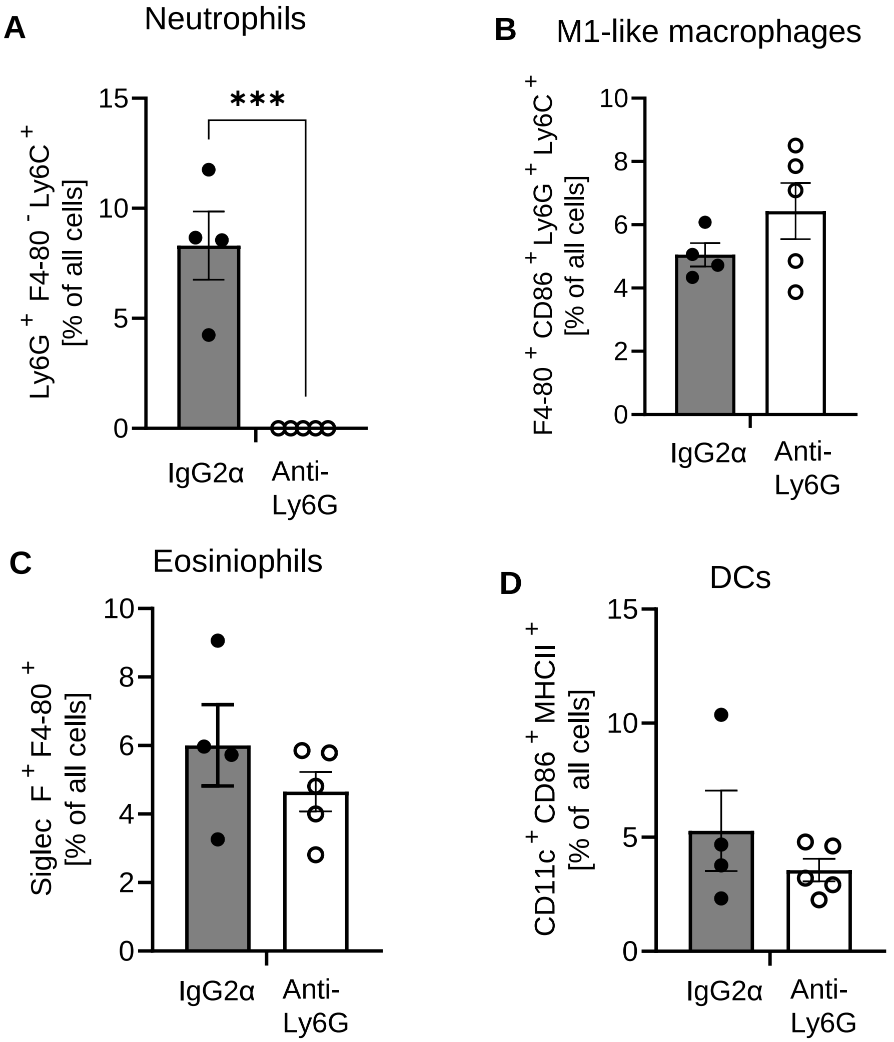

Figure S5: In vivo assay for phagocytosis of pHrodo zymosan.
(A-D) Quantification of the MELC analysis for pHrodo^{+} neutrophils (A), M1-like macrphages (B), eosinophils (C) and DCs (D) in paws 6 hours after zymosan-pHrodo injection. Data are shown as the mean ± SEM (n= 4-5); multiple unpaired t-test *** P<0.001.

Figure S6

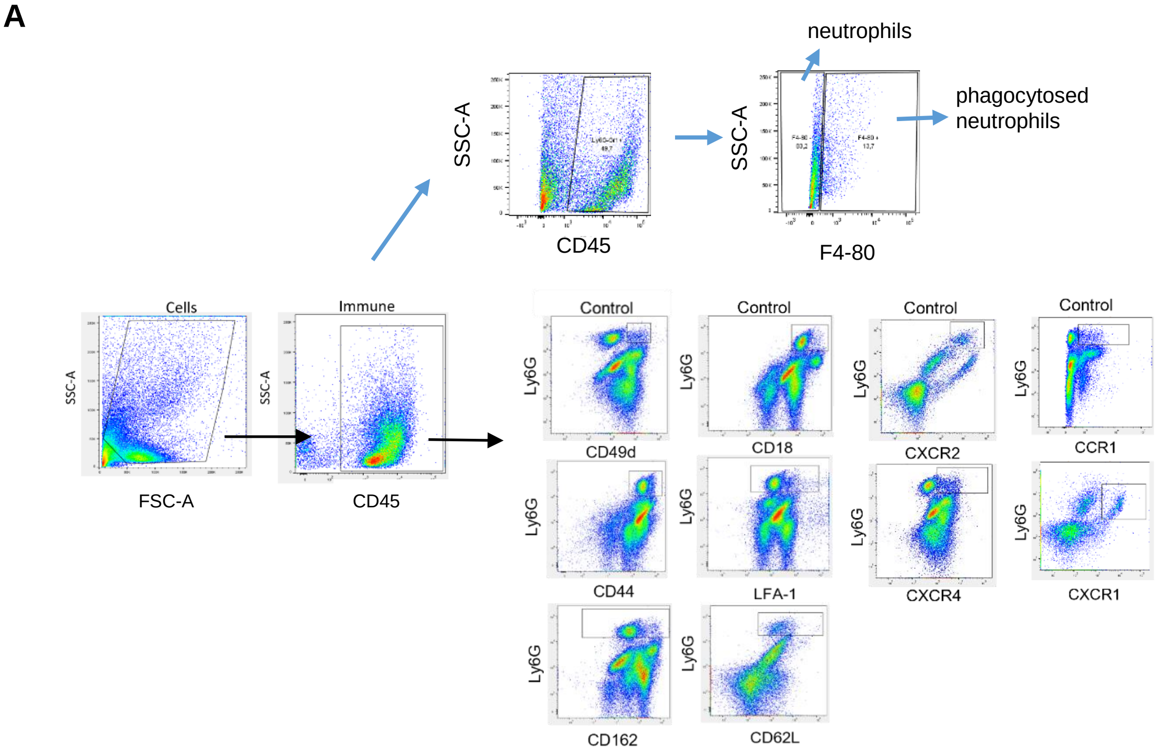

B

Name	P-value	Adjusted p-value	Odds Ratio	Combined score
* Tubulin Binding (GO:0015631)	0.00003145	0.003873	4.57	47.42
Ubiquitin-Like Protein Ligase Binding (GO:0044389)	0.00004272	0.003873	4.80	48.33
* Microtubule Binding (GO:0008017)	0.00005781	0.003873	5.10	49.73
Cyclin-Dependent Protein Serine/Threonine Kinase Regulator Activity (GO:0016538)	0.0005851	0.02440	11.57	86.13
Ubiquitin Protein Ligase Binding (GO:0031625)	0.0006069	0.02440	4.10	30.34

* matching proteins in network:
GO:0015631 Tubulin binding: Kif11, Strbp, Stmn1, Kif22, Kif15, Dlgap5, Spag5, Kif4, Ska1, Cenpe, Mast2, Pdcd5, Knstrn, Arl4c,Racgap1,Tpx2, Dnm1l
GO:0008017 Microtubule binding: Kif11, Strbp, Kif22, Kif15, Dlgap5, Spag5, Kif4, Ska1, Cenpe, Mast2, Knstrn, Racgap1, Tpx2, Dnm1l

Figure S6: Cytoskeletal functions are disrupte in GPR40 deficient neutrophils. **(A)** FACS gating strategy for neutrophils in inflammed paws (upper panel, blue arrows) 24 hours after zymosan injection and for neutrophils in blood from naive wild type mice (lower panel) for receptors known to be involved in neutrophil recruitment to inflammed tissue. **(B)** EnrichR molecular function analysis of differential mRNA expression levels in neutrophils from GPR40-knockout mice as compared to wildtype mice.

Figure S7

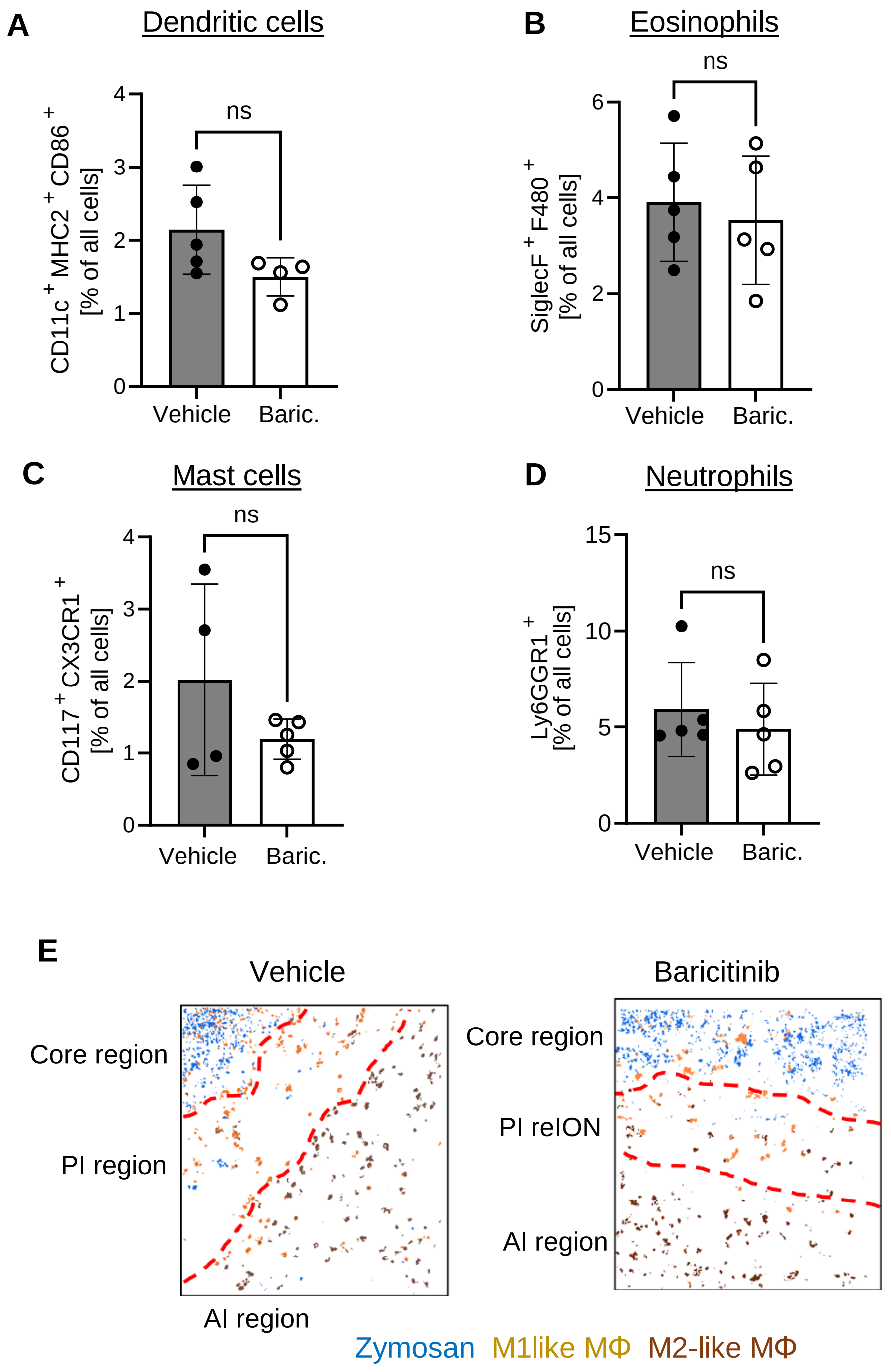

Figure S7: MELC analysis of baricitinib effect on the numbers of immune cells in inflamed paws.

(A-D) MELC analysis of the number of dendritic cells (A), eosinophils (B), mast cells (C) and neutrophils (D) in paws from vehicle or baricitinib treated mice 24 hours after zymosan-injection. Data are shown as the mean ± SEM (n=4-5); one-tailed unpaired t-test. ns, not significant. **(E)** Representative cluster images showing the inflammatory regions in paws from vehicle or baricitinib treated mice 24 hours after zymosan-injection.The red dotted lines depict the borders between the regions.

Table S8

Table S7: PI region sizes and resolution scores for zymosan-induced hyperalgesia

**Treatment**	**Size of PI region** (rel. distance zymosan to M2)	**Score 1*** days under baseline (Δ control vs. treatment)	**Score 2**** per day ΔPWL control vs. treatment	**Resolution score** (Sum Score 1+2)
Eosinophil depletion	36	-2	-2	-4
Meloxicam	30	-2	0	-2
G2A knockout	50	3	2	5
GPR40 knockout	77	0	0	0
Neutrophil depletion	88	0	0	0
Baricitinib	90	0	0	0
Mast cell deficiency	160	-2	-1	-3
TP knockout	60	0	1	1

Score 1*: One Way ANOVA/Bonferroni; ΔPWL vs. baseline
Score 2: *Two Way ANOVA/Bonferroni; ΔPWL control vs. treatment
